# The modulation of MiR-155 and MiR-23a manipulates *Klebsiella pneumoniae* Adhesion on Human pulmonary Epithelial cells via Integrin α5β1 Signaling

**DOI:** 10.1038/srep31918

**Published:** 2016-08-18

**Authors:** Yan Teng, Junming Miao, Xiaofei Shen, Xiaolong Yang, Xinyuan Wang, Laibin Ren, Xiaoying Wang, Junli Chen, Jingyu Li, Shanze Chen, Yi Wang, Ning Huang

**Affiliations:** 1 Research Unit of Infection and Immunity, Department of Pathophysiology, West China College of Basic and Forensic Medicine, Sichuan University, Chengdu 610041, China

## Abstract

Micro-RNAs (miRNAs) critically regulate several host defense mechanisms, but their roles in the bacteria-epithelium interplay remain unclear. Our results displayed that the expression of miR-155 and miR-23a were down-regulated in *K*. *pneumoniae*-infected pulmonary epithelial cells. The elevated bacterial adhesion on A549 cells followed the enhancement of the cellular levels of these two miRNAs. Meanwhile, a mechanistic study demonstrated that miR-155 promoted integrin α5β1 function and resulted in the increased actin polymerization. Moreover, a non-histone nuclear protein, high mobility group nucleosomal-binding domain 2 (HMGN2) served as the potential target of miR-155 and miR-23a to regulate the integrin α5β1 expression and *K*. *pneumoniae* adhesion. Furthermore, the expression of a known integrin transcription suppressor-Nuclear Factor-I (NFI) was also repressed by miR-155, which paralleled with its chromatin location in the promoter regions of integrin α5 and β1. These results uncover novel links between miRNAs and integrin function to regulate bacterial adhesion, indicating a potential mechanism of host cell autonomous immune response to *K*. *pneumoniae* infection.

*Klebsiella pneumoniae* (*K*. *pneumoniae*) is an opportunistic pathogen which originally resides in the intestine and penetrates epithelium to cause several hospital-acquired infectious diseases including urinary tract infection and pneumonia^1^. Most recent studies demonstrated that *K*. *pneumoniae* invaded intestinal epithelial cells through intracellular pathway where Rho GTPase and PI3K/Akt-dependent signaling were required^2^. However, how *K*. *pneumoniae* invades pulmonary epithelium and how this pathogen stimulates host autonomous immunity have not been completely unveiled.

The integrin family that contains 24 different heterodimeric proteins formed by 18α- and 8β- subunits, has been shown to precipitate in various cellular responses including bacterial internalization^3^,^4^. As integrins lack enzymatic activities, other signaling complexes such as Rho GTPases family members are involved in the signal transduction on the cytoplasmic side to accomplish numbers of downstream effects^5^,^6^. On one hand, integrin engagement is required for various steps of leukocyte-mediated pathogen clearance including chemotaxis^7^, pathogen-host cell contact sensation^8^, cell spreading and attachment^9^,^10^ and eventual ingestion of pathogens or antimicrobial agents release^11^,^12^. On the other hand, in non-professional phagocytes such as epithelial cells, numerous pathogens directly or indirectly associate with integrin through bacterial adhesive proteins to establish bacteria-host cell contact and facilitate pathogen internalization where actin cytoskeleton dynamics are subverted^3^. Particularly, β1 integrin, an extracellular matrix protein-fibronectin receptor, has been shown as the primary target of numerous invading pathogens, including *Orientiatsutsugamushi*^13^, *Yersinia enterocolitica*^14^ and *Staphylococcus aureus*^15^. Moreover, the inhibition of integrin function by either RNAi or integrin specific antibody has been proven to reduce *Staphylococcus aureus* internalization in mast cells^15^,^16^, suggesting the therapeutic potential of developing antagonistic monoclonal antibodies or small molecules targeting integrins in treating infectious and inflammatory diseases^17^.

Pathogen-induced immune responses and self-defense mechanisms rely on the accurate but swift reprogramming of the host gene expression, where miRNA as one group of epigenetic factors is shown to fine-tune this process^18^. Among the expanding profile of immune-responsive miRNAs, miR-155 and miR-23a are of particular interests based on their functions in host immunity^19^,^20^,^21^. MiR-155 is processed from an exon of the noncoding RNA from *bic*locus^22^. Multiple lines of evidence have shown that miR-155 appears to be sentinels for immune responses in active B cells^23^, T cells^24^,^25^, macrophages and dendritic cells (DCs)^26^,^27^. Meanwhile, miR-23 which is generated from the transcripts of miR-23 ~ 27 ~ 24 clusters was primarily focused by its roles in tumorigenesis^28^,^29^. The emerging studies have reported miR-23 functions as an antiviral factor against PRRSV infection^20^ and also participates in regulating T cell differentiation under immune challenge^21^, strongly indicating its essential role in adaptive immunity. However, the functions of these two miRNAs in the autonomous immunity regulation of epithelial cells are still largely unknown.

In the present study, the potential mechanism of miR-155 and miR-23a in modulating *K*. *pneumoniae* infection in pulmonary epithelial cells was investigated. Our data exhibited that the expression of miR-155 and miR-23a was surprisingly decreased after *K*. *pneumoniae* adhesion. The enhanced expression of these two miRNAs accelerated the bacterial adhesion on A549 cells, while the block of miR-155 level reversed the result. The mechanistic study demonstrated that miR-155 promoted integrin α5β1 function and resulted in the increased actin polymerization. HMGN2 served as the potential target of miR-155 and miR-23a to regulate the integrin expression and *K*. *pneumoniae* adhesion. Alternatively, miR-155 targeted Nuclear Factor-I (NFI) family where NFIB is a known integrin transcription suppressor^30^. The block of miR-155 level of A549 cells enhanced the global level of NFI expression as well as it occupancy at integrin α5/β1 promoter regions. The pharmacological inhibition of integrin pathway or actin polymerization compromised *K*. *pneumoniae* adhesion which was induced by the overexpression of miR-155 or miR-23a respectively. Our results reveal a novel link between miRNAs and integrin/Rac1-dependent actin dynamics regulation in pulmonary epithelial cells, which might be utilized by host cell autonomous immunity to impede *K*. *pneumoniae* adhesion.

## Results

### MiR-155 and miR-23a are down-regulated in *K*. *pneumoniae* infected pulmonary epithelial cells and promote *K*. *pneumoniae* adhesion

**Figure 1:**
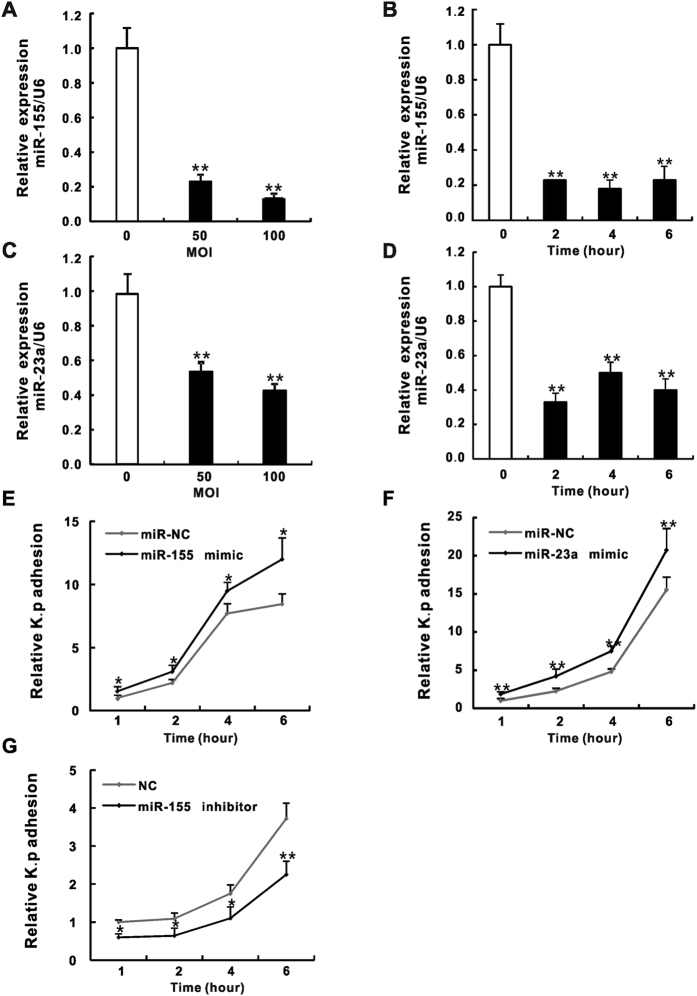
MiR-155 and miR-23a expression were down-regulated in *K*. *pneumoniae* infected A549 cells and promoted *K*. *pneumoniae* adhesion. A549 cells were exposed to increasing doses of *K*. *pneumoniae* (MOI = 0, 50, 100) for 2 hours, or fixed amount of bacteria (MOI = 100) at indicated time points (0 hr, 2 hr, 4 hr and 6 hr). The expression levels of miR-155 (**A**,**B**) and miR-23a (**C**,**D**) were examined by RT-qPCR. The Relative expression was normalized to U6 and then converted to the fold change over uninfected. A549 cells were transfected with miR-155 mimic (**E**), miR-23a mimic (**F**), miR-155 inhibitor (**G**) and according negative controls (miR-NC or NC) for 24 hours prior to 100 MOI of bacterial exposure. The relative *K*. *pneumoniae* adhesion at indicated time points were determined by colony counts. Relative *K*. *p* adhesion was represented after the normalization to 1 hour bacterial adhesion of miR-NC or NC. (Data are the mean ± SD and represent three individual experiments. *p < 0.05, **p < 0.01 compared with *K*. *pneumoniae* uninfected, miR-NC or NC).

To investigate roles of miR-155 and miR-23a in pulmonary epithelial cells during bacterial infection, we conducted a quantitative RT-qPCR assay to analyze the expression of these two miRNAs in human alveolar type II epithelial cell line A549 and/or bronchial epithelial cell line HBE16 that were exposed to *K*. *pneumoniae*. To our surprise, the expression of miR-155 (Fig. 1A,B and S1A) and miR-23a (Fig. 1C,D) were both significantly down-regulated upon bacterial infection where the dosage (the multiplicity of infection (MOI) of *K*. *pneumoniae* was from 50 to 100) and time (the infection time was from 2 to 6 hours) dependences were not observed. In addition, the expression of miR-155 precursor-primary miR-155 (pri-miR-155) in *K*. *pneumoniae* treated A549 and HBE16 cells displayed the similar trends as that of miR-155 except for the partial recovery after 2 hours bacterial exposure (Fig. S1B,C). These results seemed unexpected especially for miR-155, as multiple studies have shown that the expression of miR-155 might be induced by bacterial infection^31^ or pro-inflammatory stimulation by using lipopolysaccharide (LPS), Tumor Necrosis Factor α (TNF-α), interferon (IFN) and polyribocytidylic acid (poly IC) in phagocytes^26^,^32^. To reconcile such controversy, we further confirmed our results by showing the induction of miR-155 in both A549 cells and RAW264.7 macrophage treated by LPS (Fig. S1D). However, the exposure of A549 cells to *K*. *pneumoniae* caused significant reduction of miR-155 expression compared with still elevated miR-155 levels in bacteria stimulated RAW264.7 (Fig. S1E). Thus, our data indicate different regulatory mechanisms of miR-155 expression responding to pathogen stimuli residing in pulmonary epithelial cells and phagocytes. To gain insights into how miR-155 and miR-23a influence *K*. *pneumoniae* infection, the bacterial adhesion assay was performed. Cells were transfected with mimic or inhibitor oligoribonucleotides of miR-155 or miR-23a respectively prior to different time lengths of *K*. *pneumoniae* exposure. The adhesion efficiency evaluated by colony counting showed that transfection of miR-155 or miR-23a mimic effectively increased the bacterial adhesion at all time points we checked (Figs 1E,F and S2A), whereas the miR-155 inhibitor reversed the results to its mimic (Figs 1G and S2B). Collectively, our results showed an unexpected moderation of miR-155 and miR-23a during *K*. *pneumoniae* infection of epithelial cells that potentially harnessed bacterial adhesion (see discussion).

### HMGN2 is the potential target of miR-155 and miR-23a to participate in the regulation of *K*. *pneumoniae* adhesion

**Figure 2:**
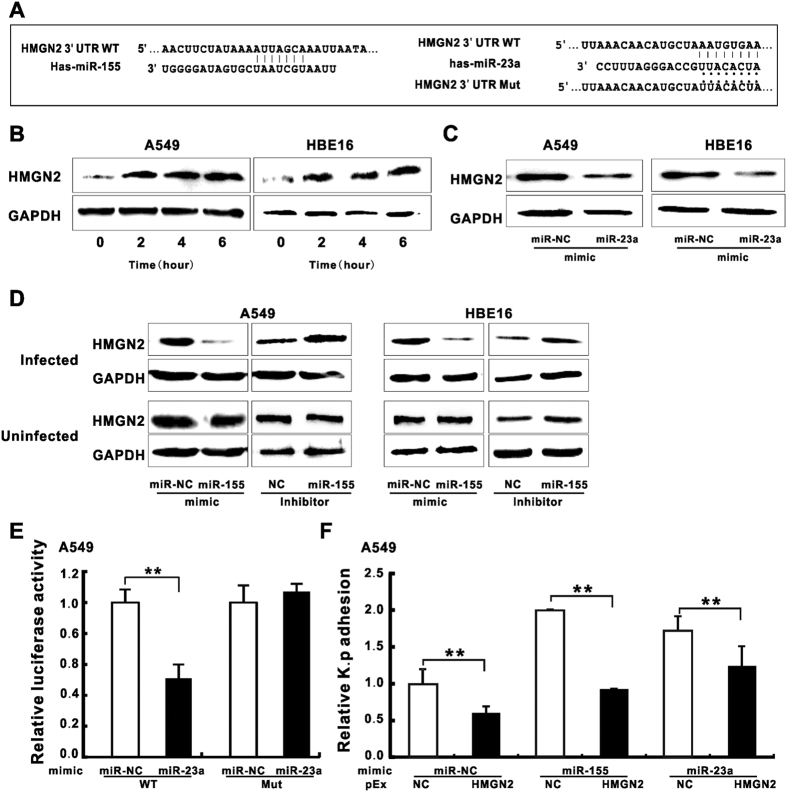
HMGN2 was the potential target of miR-155 and miR-23a to involve in regulating *K*. *pneumoniae* adhesion. (**A**) Schematic presentation of base pairing between the 3′ UTR of HMGN2 and miR-155 or miR-23a by erect likes. The mutant seed sequence of the HMGN2 3′ UTR matching miR-23a is also presented by dots. (**B**) Western blot analysis showing the change of HMGN2 protein level in *K*. *pneumoniae* infected A549 and HBE16 cells at different infection time (MOI = 100). (**C**) Western blot analysis showing the effect of miR-23a mimic on the protein expressions of HMGN2 in *K*. *pneumoniae* infected A549 or HBE16 cells (MOI = 100 for 2 hours, same as **D**–**F**). (**D**) Western blot analysis showing the effect of miR-155 mimic or inhibitor on the protein expressions of HMGN2 in *K*. *pneumoniae* infected or uninfected cells. (**E**) A549 cells were co-transfected with miR-23a mimic or miR-NC along with HMGN2 3′ UTR wild-type (WT) or mutant (MUT) reporter. Luciferase activity was measured 24 hr after transfection. (**F**) The relative *K*. *pneumoniae* adhesion in A549 cells co-transfected with pEx-HMGN2 and miR-155 or miR-23a mimic. (Data are the mean ± SD and represent three individual experiments. **p < 0.01 compared with miR-NC and pEx-NC cotransfection).

We then applied an online algorithmfor miRNA target prediction (TargetScan) to identify the putative binding sequences for miR-155 or miR-23a in 3′ UTR of HMGN2 mRNA (Fig. 2A). Of particular interest, our previous study has demonstrated that HMGN2 served as an anti-bacterial peptide^33^ and the knockdown of HMGN2 correlated with enhanced bacterial internalization (Wang, in press), which resembled the effects of forced expression of miR-155 or miR-23a. We therefore hypothesized that HMGN2 level is targeted by miR-155 and/or miR-23a in un-infected epithelial cells, whereas the exposure to *K*. *pneumoniae* de-represses its expression. As we expected, we found the elevated HMGN2 expression at different time points after *K*. *pneumoniae* infection in both A549 and HBE16 cells (Fig. 2B), regardless of its mRNA level (Fig. S3A). Previous study suggested HMGN2 was regulated by miR-23a^34^. In our experiment, HMGN2 was significantly suppressed by mimics of miR-23a (Fig. 2C) and miR-155 (Fig. 2D upper panel) during *K*. *pneumoniae* infection, while the miR-155 inhibitor displayed the opposite effect in both cell lines. Strikingly, the modulation of HMGN2 by miR-155 displayed an infection-dependent manner as our result showed the cellular level of HMGN2 responded to miR-155 mimic or inhibitor specifically during *K*. *pneumoniae* infection compared with un-infected group (Fig. 2D lower panel). To further inspect the targeting of HMGN2 by miRNAs, we performed luciferase reporter assay where the luciferase reporter was cloned with the 3′ UTR of HMGN2 transcript containing miR-23a and miR-155 specific targeting sequences. Consistent with previous result^34^, we observed the marked reduction of the luciferase activity in the wild type reporter but not the mutant by transfecting miR-23a mimic (Fig. 2E), reinforcing the direct targeting of HMGN2 mRNA by miR-23a. However, neither the HMGN2 mRNA level (Fig. S3B) nor the luciferase activity of the reporter construct (Fig. S3C) was responsive to miR-155 mimic or inhibitor implying the indirect regulation of miR-155 on HMGN2. Moreover, it was less likely that miR-155 regulated HMGN2 protein level via the proteasome-dependent mechanism as the administration of proteasome-specific inhibitor MG132 did not affect HMGN2 protein levels (Fig. S3D). To test the involvement of HMGN2 in miRNA-mediated bacterial adhesion regulation, we co-transfected HMGN2 vector (pEx-HMGN2) with mimics of miR-155 or miR-23a prior to *K*. *pneumoniae* exposure and measured bacterial adhesion efficiency. Our result showed that the transfection of HMGN2 vectors significantly increased its protein levels (Fig. S3E) and in turn counteracted the bacterial adhesion that was induced by miR-155 or miR-23a mimic (Fig. 2F), suggesting HMGN2 participates in miR-155 and miR-23a-mediated *K*. *pneumoniae* infection.

### MiR-155 manipulates integrin α5β1/Rac1 pathway function and actin polymerization in *K*. *pneumoniae* infected A549 cells

**Figure 3:**
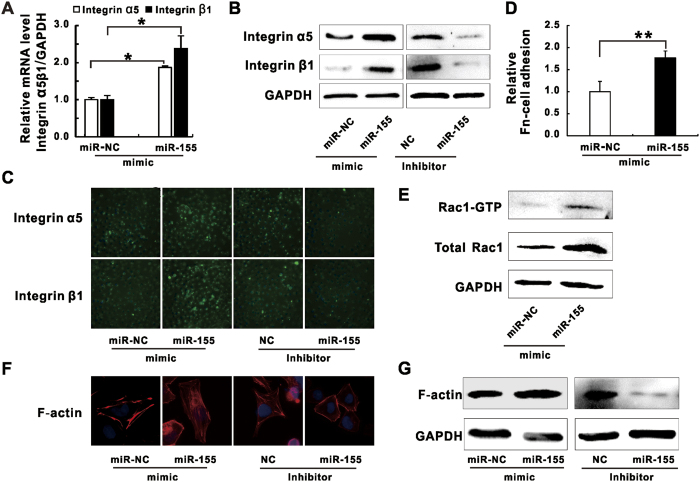
MiR-155 promoted integrin/Rac1 activity during *K*. *pneumoniae* infection. A549 cells were transfected with miR-155 mimic and according negative controls (miR-NC), or miR-155 inhibitor and according negative controls (NC) for 24 hours prior to 100 MOI of *K*. *pneumoniae* infection. (**A**) RT-qPCR analysis showing the mRNA levels of integrin α5 and β1. (**B**) Western blot analysis showing the expressions of integrin α5, integrin β1. (**C**) Microscopic images displaying the expressions of integrin α5 and β1 (green fluorescence, 40×). Blue fluorescence represented the nucleus staining by DAPI. (**D**) Fibronectin (FN)-cell Adhesion assay was performed to evaluate the effect of miR-155 mimic on uninfected A549 cells to associate with fibronectin coated plates. (**E**) Western blot analysis showing the pull-downed active form of Rac1 (Rac1-GTP) and total Rac1 levels. (**F**) Microscopic images displaying the membrane ruffles formed by polymerized F-actin (F-actin: red fluorescence, DAPI: blue fluorescence, 100×). (**G**) Western blot analysis showing the expressions of F-actin. (Data are the mean ± SD and represent three individual experiments. *p < 0.05, **p < 0.01 compared with miR-NC).

It has been well studied that many pathogenic bacteria enter into non-phagocytic host cells by exploiting integrin-mediated signaling pathway^3^. To investigate whether miR-155 promotes *K*. *pneumoniae* adhesion by manipulating integrin function, we examined the expression of integrin α5 and β1 in epithelial cells transfected with miR-155 mimic or inhibitor. Our results showed miR-155 mimic enhanced both mRNA and protein levels of these two integrins in A549 (Fig. 3A,B) and HBE16 cells (Fig. S4A,B) while the inhibitor suppressed protein expression. In accordance with immunoblotting results, our fluorescence microscopy data displayed enhanced or reduced fluorescence densities of integrin α5 and β1 in A549 cells transfected with miR-155 mimic or inhibitor respectively (Fig. 3C). Furthermore, cell surface-expressed integrins reflecting integrin functions were measured by fibronectin (FN)-cell binding assay^35^ and the increased cell association on FN-coated plates were observed for miR-155 mimic transfected A549 (Fig. 3D) and HBE16 cells (Fig. S4C). As integrin-mediated pathogens internalization requires intracellular signal transduction, including Rho family GTPases activation and actin cytoskeleton rearrangement^5^, we sought to check whether miR-155 stimulated Rho GTPases activities and actin dynamic during infection. Firstly, our results showed the expression and the activity of Rho GTP family member Rac1 were enhanced by transfection of miR-155 mimic (Figs 3E and S4D). Moreover, the increase and the decrease of actin expression as well as membrane ruffles formation of polymerized actin filaments (F-actin) followed miR-155 mimic and inhibitor treatment were also detected in A549 (Fig. 3F,G) and HBE16 cells (Fig. S4E). Thus, our results demonstrated miR-155 stimulated integrin α5 and β1 functions as well as activated Rho GTPase Rac1 activity, which correlated with induced actin polymerization during *K*. *pneumoniae* infection.

### HMGN2 is involved in miR-155-mediated integrin/Rac1 activation in A549 cells during *K*. *pneumoniae* infection

**Figure 4:**
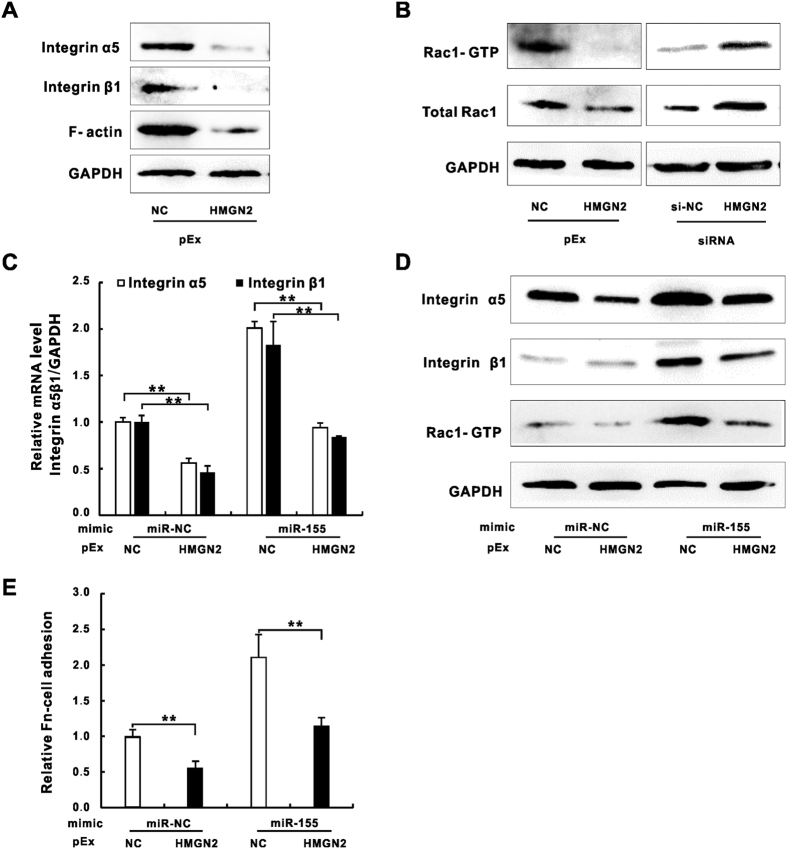
HMGN2 was involved in miR-155 mediated Integrin/Rac1 regulation during *K*. *pneumoniae* infection. Western blot analysis showing the expressions of integrin α5, integrin β1 and F-actin (**A**) or Rac1-GTP and the total Rac1 (**B**) in A549 cells transfected with pEx-HMGN2 and/or siRNA-HMGN2 prior to *K*. *pneumoniae* exposure (MOI = 100 for 2 hours, same as C and D). The relative mRNA level of integrin α5 and β1 (**C**), the protein level of integrin α5, integrin β1 and Rac1-GTP (**D**) of A549 cells co-transfected with pEx-HMGN2 and miR-155 mimic prior to *K*. *pneumoniae* exposure. (**E**) The relative FN-cell adhesion of uninfected A549 cells transfected as (**C**,**D**). (Data are the mean ± SD and represent three individual experiments. **p < 0.01 normalized with miR-NC and pEx-NC co-transfection).

HMGN2 has been well studied for its regulatory roles in general chromatin functions by altering nucleosome structures^36^,^37^. Although, the deletion of HMGN2 gene alone might not induce dramatic changes of genome wide transcription profile^38^, the encoded protein was reported to serve as a transcriptional modulator for a certain subset of genes involved in Wnt/β catenin signaling and Jak2/Stat5a pathways^39^,^40^. Our previous studies demonstrated that HMGN2 plays critical roles in regulating LPS-mediated antimicrobial peptide β-defensin-2 (HBD-2) expression in A549 cells and a mice model^41^,^42^, suggesting its functions in regulating gene expression related to host innate immune response. To further determine roles of HMGN2 in integrin pathways, we examined the integrin, Rac1 and actin expression in HMGN2 overexpressed or knockdown A549 cells. The immunoblotting assay showed the down-regulation of integrin levels that were followed by the reduced actin expression in HMGN2 overexpressed cells (Fig. 4A). Moreover, the enhanced expression of HMGN2 decreased Rac1 level and its GTPase activity while silencing HMGN2 by siRNA reversed these results (Fig. 4B). HMGN2 displayed regulatory functions of bacterial internalization both *in vivo* and *in vitro*^41^,^42^. We therefore sought to ask whether HMGN2 participated in miR-155 regulation of integrin and Rac1 function by co-transfecting A549 cells with miR-155 mimic and HMGN2 vector prior to *K*. *pneumoniae* exposure. Our result showed while miR-155 mimic alone elevated integrin α5 and β1 mRNA by two folds, the overexpression of HMGN2 attenuated the stimulation of integrin transcription (Fig. 4C). The immunoblotting results consisted with RT-qPCR data showing miR-155-mediated elevation of integrin α5 and β1 protein levels and Rac1 activity were also compromised by overexpression of HMGN2 (Fig. 4D). Moreover, HMGN2 antagonized the stimulating effect of miR-155 mimic on integrin α5 and β1 activities in FN-cell the adhesion assay (Fig. 4E). Taken together, our results demonstrated that HMGN2 contributed to the miR-155-mediated integrin α5 and β1 function of A549 cells during *K*. *pneumoniae* infection.

### MiR-155 inhibits a known integrin transcription suppressor NFI during *K*. *pneumoniae* infection

**Figure 5:**
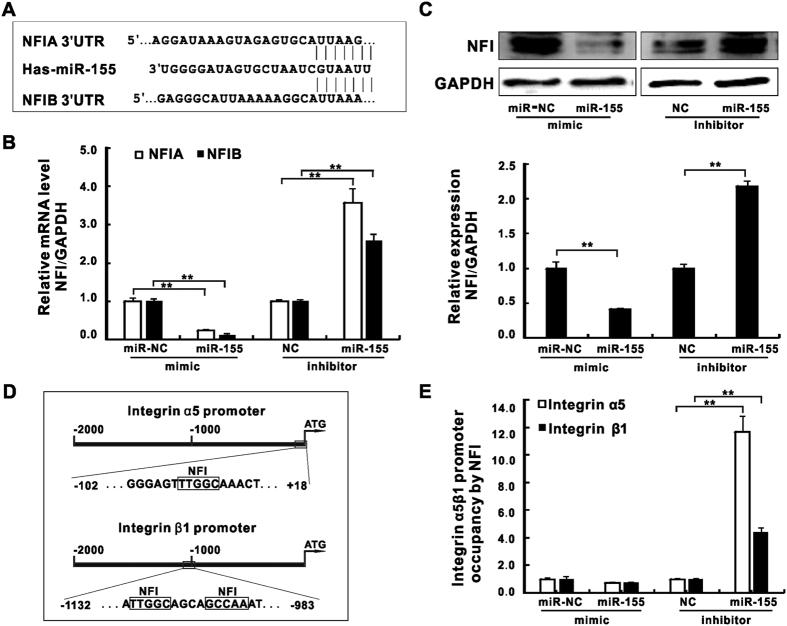
MiR-155 inhibited a known integrin transcription suppressor NFI during *K*. *pneumoniae* infection. (**A**) Schematic presentation of base pairing between miR-155 and the 3′ UTR of NFIA or NFIB by erect lines. The mRNA expressions (**B**) and the protein levels (**C**) of NFIA and NFIB in A549 cells transfected with miR-155 mimic or inhibitor prior to *K*. *pneumoniae* exposure (MOI = 100 for 2 hours). (**D**) The schematic diagram of NFI binding motifs (TTGGC, GCCAA) on promoters of integrin α5 or β1 (−2000 bp to TSS site ATG). The primers for ChIp assay were designed for about 60–70 bp up-stream or down-stream of NFI binding motifs (−102 to +18 bp on α5 promoter, −1132 and −983 bp on β1 promoter). (**E**) ChIp assay showingthe NFI recruitment at the desired regions shown in (**D**) under the same condition as (**B**,**C**), the relative occupancy is the ratio of immunoprecipitated NFI to input DNA. (Data are the mean ± SD and represent three individual experiments. **p < 0.01 compared with miR-NC or NC).

Besides HMGN2, our miRNA target-prediction analysis also identified two Nuclear Factor I (NFI) family members, NFIA and NFIB, as potential targets of miR-155 (Fig. 5A). NFI transcription factors are a group of site-specific DNA-binding proteins that have been well studied in various biological processes^43^. It has been shown that the *bona fide* NFI binding sites reside in promoter regions of both integrin α5 and β1 as they are required for potent suppression of integrin α5 transcription^30^,^44^. To inspect if miR-155 regulates integrin functions through targeting NFI family members, we firstly analyzed mRNA expression of NFIA and NFIB in A549 cells transfected with miR-155 mimic or inhibitor respectively (Fig. 5B). As expected, miR-155 mimic caused nearly 80% reduction of mRNA levels of NFIA and B while its inhibitor resulted in more than two folds of transcription induction. Consistent with RT-qPCR results, NFI protein levels were reduced by miR-155 mimic but increased by its inhibitor (Fig. 5C). It has been shown that NFI binds to highly specified DNA motifs (TTGGC and GCCAA)^45^. To further examine if miR-155 altered the recruitment of NFI at its intrinsic binding sites in integrin promoters, we performed a Chromatin Immunoprecipitation assay (ChIP) (Fig. 5D). According to our ChIP data, despite the modest reduction of NFI recruitment at both integrin α5 and β1 promoters by miR-155 mimic transfection, the inhibition of miR-155 resulted in enhanced NFI enrichment by 4 to 12 folds respectively (Fig. 5E). Our results indicate a likelihood that besides HMGN2 targeting, miR-155 might also regulate integrin function by manipulating the expression and chromatin location of another integrin transcription suppressor NFI during *K*. *pneumoniae* infection.

### Pharmacological inhibition of integrin/Rac1 pathway and actin polymerization partially block *K*. *pneumoniae* adhesion induced by miR-155 and miR-23a

**Figure 6:**
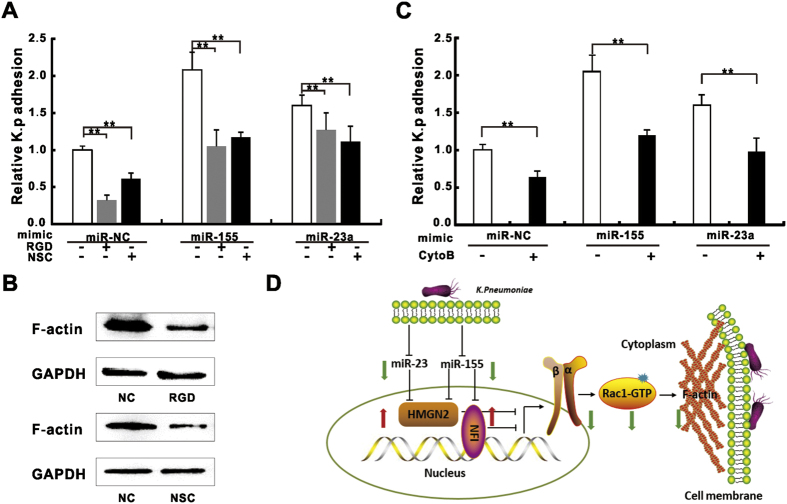
Pharmacological inhibition of Integrin/Rac1 and acting polymerization partially blocked miR-155 and miR-23a induced *K*. *pneumoniae* adhesion. *K*. *pneumoniae* adhesion was measured on A549 cells transfected with miRNA mimics and then treated with RGD (50 nM, 24 hours) or NSC23766 (NSC, 50 μM, 2 hours) (**A**) or cytochalasin (**B**) (CytoB, 10 μM, 2 hours) (**C**) prior to *K*. *pneumoniae exposure* (MOI = 100 for 2 hours). Untreated miR-NC was defined as 100% of relative adhesion. (**B**) Western blot analysis showing the expressions of F-actin in A549 cells treated with RGD or NSC23766 under the same condition as (**A**). (Data are the mean ± SD and represent three individual experiments. **p < 0.01). (**D**) The schematic diagram depicting miR-155 and miR23a pathway: miRNAs promote intergrin α5β1 and Rac1 activities by targeting negative transcriptional regulators of integrin -HMGN2 and NFI, and result in actin polymerization (black solid lines). The exposure of *K*. *pneumoniae* causes the active reduction of these two miRNAs, which subsequently de-represses HMGN2 and NFI to inhibit integrin/Rac1 function and slows actin polymerization (green and red arrows).

To further confirm the involvement of integrin function in miR-155 or miR-23a-mediated *K*. *pneumoniae* adhesion regulation, we applied integrin inhibitor-RGD tri-peptide, and Rac1 GTPase specific inhibitor-NSC23766 to block integrin and Rac1 signaling during *K*. *pneumoniae* infection. We observed that administration of RGD and NSC23766 alone significantly decreased bacterial adhesion rate (Fig. 6A), indicating the engagement of integrin/Rac1 pathway is required for the regulation of the *K*. *pneumoniae* adhesion. The pre-treatment of A549 cells with RGD or NSC23766 significantly abolished *K*. *pneumoniae* adhesion that was promoted by miR-155 or miR-23a respectively (Fig. 6A), suggesting the involvement of miRNAs in this process. Additionally, we also observed the administration of these two inhibitors significantly decreased the expression of actin (Fig. 6B), which is considered a major downstream target of integrin^5^. Previous study showed that inhibiting actin polymerization by cytochalasin B blocked pathogen induced cytoskeleton rearrangement and impede microbe up-take^46^. Consistently, the application of cytochalasin B attenuated the enhancement of *K*. *pneumoniae* adhesion by either miRNAs (Fig. 6C). Taken together, we concluded that integrin/Rac1 pathway as well as actin polymerization were involved in miR-155 and miR-23a-mediated *K*. *pneumoniae* adhesion regulation (Fig. 6D; See discussion).

## Discussion

MiRNAs have emerged as novel posttranscriptional regulators to participate in plenty of cellular processes, such as cell proliferation, differentiation, apoptosis and immune response^18^. Nonetheless, the underlying molecular mechanism involved in the miRNA-mediated pathogen-host immune regulation remains incomprehensive. Our study demonstrated a potential mechanism utilized by pulmonary epithelial cells during *K*. *pneumoniae* infection: host cells actively down-regulate the cellular levels of miR-155 and miR-23a which target non-histone nuclear factors HMGN2 and/or NFI. The de-repression of HMGN2 and NFI as negative modulators of integrin α5 and β1 in turn weaken the activation of integrin/Rac1 signaling and actin cytoskeleton re-arrangement which are required for *K*. *pneumoniae* adhesion (Fig. 6D).

The cellular miRNAs expression is under sophisticated modulation, requiring precise control of outside stimulation to inside signal transduction and recruitment of transcription factors to the promoter area of miRNAs genes^18^. Our data showed that miR-155 and miR-23a expression were dramatically decreased in A549 and HBE16 cells after *K*. *pneumoniae* infection (Figs 1A–D and S1A), which seemed to be controversial to the previous reports. It has been shown that miR-155 is induced by several pro-inflammatory agents such as LPS, IFN, poly IC or TNF-a in monocytes, macrophages and dendritic cells^26^,^32^, however, it is also down-regulated by anti-inflammatory cytokines including IL-10, IL-4 and TGF-β in monocytes, fibroblast-like cells and lung fibroblasts^47^,^48^,^49^,^50^. Meanwhile, miR-23a was found to be repressed by NF-κB member p65 and PML-RARA fusion protein in human leukemic Jurkat cells^51^ and myeloid tumor cells^52^. All of these lines of evidence indicate negative regulations of miRNA expression regulation for varying beneficial effects. Specifically, anti-inflammatory agent IL-10 inhibits miR-155 expression in LPS activated macrophages to slow cellular inflammatory response^48^,^50^,^53^. This effect was also discovered in *Orientiatsutsugamushi* infected macrophages^54^, LPS stimulated B cells^55^ and a murine model of *Borreliaburgdorferi*-induced Lyme arthritis and carditis^56^. Another canonic anti-inflammatory cytokine IL-4 was also found to negatively regulate miR-155 in fibroblast-like cells^47^. Similarly, miR-23a is commonly down-regulated in lymphoid tumor cells^57^, and this modulation was found to de-repress glutaminase (GLS) expression for tumor cell proliferation and survival under elevated glutamine consumption condition^51^. It is possible that the various virulent agents of *K*. *pneumoniae* that stimulate different pathogen sensor molecules like Toll-like receptors in host cells can result in diversified downstream signal cascades^58^. Our data from A549 cells and RAW264.7 macrophages that were stimulated by *K*. *pneumoniae* or LPS respectively demonstrate that LPS induce miR-155 expression in both cells (Fig. S1D). However, treating A549 cells but not RAW 264.7 with *K*. *pneumoniae* reduced the expression of this particular miRNA (Fig. S1E). These side-by-side comparisons strongly indicate that although LPS, as one of the major virulent effectors of *K*. *pneumoniae*, contributes to the induction of miR-155 levels in cells, there are other unknown inhibitory factors or signaling pathways in pulmonary epithelial cells to overcome LPS-mediated miRNA stimulation. In addition, it was well known that bacterial infections can lead to activate NF-κB pathway, which is characterized by p65/p50 translocation into nuclear^59^. As mentioned above, p65 represses miR-23a expression, rendering the possibility that *K*. *pneumonia*e infection results in the down-regulation of these two miRNAs via NF-κB pathway. Furthermore, other transcription suppressors that directly target the promoters of *bic* or a miR-23∼24∼27 cluster could also participate in the transcriptional repression of these precursor genes since the pri-miR-155 level was detected to correlate with miR-155 reduction (Fig. S1B,C).

Invasive microbes evolve highly sophisticated strategies to manipulate host molecular signaling for bacteria-host adhesion and eventual invasion across cellular membranes of non-phagocytic cells^60^. During the bacterial internalization process, the activation of integrin-mediated actin polymerization is important and has been well documented in numerous cases of bacterial infection^5^. Of a note, inhibiting bacterial-induced integrin activity and actin polymerization directly attenuates the internalization of bacteria^60^. In the present study, we used mimic and/or inhibitor of miRNAs to demonstrate that miR-155 and miR-23a might stimulate *K*. *pneumoniae* adhesion in pulmonary epithelial cells (Figs 1E–G and S2A,B) by targeting two negative transcriptional modulators of integrins-HMGN2 (Fig. 2C,D) and NFI (Fig. 5C). And this subsequently led to the change of host cell cytoskeleton dynamics (Fig. 6B). However, our results seemed to be a little counterintuitive that in the cells treated with *K*. *pneumoniae* alone where the endogenous levels of miRNAs had already been brought down (Fig. 1A–D), the function of integrins and Rho GTPases might supposedly be turned on^60^. Given that miR-155 and miR-23a facilitated *K*. *pneumoniae* adhesion (Fig. 1E,F), we postulated it was possible that host cells might utilize unknown strategies to restrict their cellular expression in order to neutralize integrin engagement and impede acute internalization of pathogens. We firstly showed that the dramatically increased cellular miR-155 level by the transfection of mimic (Fig. S2C) significantly suppressed the expression of HMGN2 (Fig. 2D) and NFI (Fig. 5C) as well as NFI localization of integrin promoters (Fig. 5E). As a result, the integrin function and actin cytoskeleton re-arrangement in host cells were largely de-repressed (Fig. 3B,E,G) while *K*. *pneumoniae* adhesion efficiency was promoted (Figs 1E and S2A). Meanwhile, the already down-regulated miR-155 level in the infected epithelial cells was further decreased by inhibitor transfection (50–80%) (Fig. S2D), which slowed the *K*. *pneumoniae* adhesion rate (Figs 1G and S2B), indicating that the natural reduction of the endogenous miR-155 by bacterial stimulation was not sufficient to reverse integrin activation and F-actin formation. In addition, our argument was supported by the recent study showing that miR-155 suppressed the macrophage-mediated bacterial phagocytosis and intracellular killing of *P*. *aeruginosa* by targeting Rheb^31^. Therefore, we proposed that during *K*. *pneumoniae* infection, pulmonary epithelial cells autonomously shut down the expression of miR-155 and/or miR-23a as well as downstream integrin pathway to potentially delay the bacterial invasion.

In our study, we discovered HMGN2 were under regulation of miR-155 and miR-23a, although the underlying mechanisms seemed to vary. MiR-23a was shown to directly target HMGN2 mRNA 3′ UTR by luciferase assay (Fig. 2E), while miR-155 only influenced the protein level (Fig. 2D) but not the mRNA level of HMGN2 (Fig. S3B,C) indicating the indirect regulation of miR-155 on this protein. In addition, our pharmacological results revealed miR-155 and miR-23a promoted *K*. *pneumoniae* adhesion partially through integrin function and actin polymerization by using specific inhibitor targeting integrin, Rac1 and actin polymerization (Fig. 6A–C). We also noticed that miR-155 relied on integrin/Rac1 pathway more than miR-23a did since the administration of RGD and NSC23766 caused more reduction of *K*. *pneumoniae* adhesion in miR-155 mimic transfected cells than that in miR-23a. More interestingly, HMGN2 protein level responded to miR-155 modulation only under infectious condition (Fig. 2D), suggesting an unknown infection-specific mechanism play potential roles in miR-155-mediated HMGN2 expression regulation. Moreover, previous studies have shown that HMGN2 participates in host cell innate immunity against various pathogens, both *in vitro* and *in vivo* by directly serving as small anti-infection effector or transcriptional modulator of human antimicrobial peptide β-defensin^33^,^41^,^42^. Herein, our data further suggest multiple mechanisms may participate in the miRNA-mediated and infection-dependent HMGN2-integrin-actin axis to regulate host cell autonomous immune response.

## Materials and Methods

### Chemical reagents and antibodies

RGD peptide, Fibronectin, lipopolysaccharide (*Escherichia coli* 0111:B4), Cytochalasin B, Rhodamine-conjugated phalloidin and proteasome inhibitor MG132 were obtained from Sigma-Aldrich (Shanghai, China). NSC23766 was the product of Selleck Chemicals (Shanghai, China). Rabbit monoclonal antibodies for HMGN2 was from Cell Signaling Technology Inc. (Danvers, USA). Mouse monoclonal antibody for F-actin and Rabbit monoclonal antibodies for integrin α5, integrin β1 were purchased from Abcam (Cambridge, USA). Mouse and rabbit monoclonal antibodies for GAPDH, horseradish peroxidase (HRP)-conjugated secondary antibody and FITC fluorescent-labeled secondary antibody (goat anti-rabbit IgG, green) were provided by Beyotime Institute of Biotechnology (Haimen, China). Rabbit polyclonal antibody for NFI was provided by Santa Cruz Biotechnology Inc. (Santa Cruz, CA). Rac1 activation assay kit with anti-active Rac1 (Rac1-GTP) monoclonal antibody and anti-Rac1 rabbit polyclonal antibody were provided by NewEast Biosciences (Malvern, USA).

### Microbial strains and cell culture

The *Klebsiella pneumoniae* clinical isolate was preserved in our laboratory, the strain was identified as *Klebsiella pneumoniae* by API 20E (bioMérieux, Marcy-l′Étoile, France). Bacteria were grown to logarithmic phase in Luria–Bertani (LB) broth at 37 °C. The concentration of microorganism suspensions were determined by measuring absorbance at 625 nm.

The human alveolar type II epithelial cell line (A549 cell), human bronchial epithelial cell line (HBE16 cell) and RAW264.7 macrophage were purchased from the Cell Bank of the Chinese Academic of Sciences (Shanghai, China), A549 and HBE16 cells were cultured in RPMI 1640 medium (Hyclone Thermo Scientific, Beijing, China) supplement with 10% fetal bovine serum (FuMeng Gene Co., Ltd., Shanghai, China) and antibiotics (100 U/ml penicillin and 100 μg/ml streptomycin, Beyotime, Haimen, China). RAW264.7 cells was cultured in Dulbecco’s Modified Eagle Medium (DMEM) supplemented with 10% (v/v) heat-inactivated fetal bovine serum and antibiotics (100 U/ml penicillin and 100 μg/ml streptomycin). All cells were incubated at 37 °C in humidified air with 5% CO_2._

### Plasmid Constructs

HMGN2 overexpression vector (pEx-HMGN2) containing CDS region of HMGN2 mRNA was cloned into pEx-GFP plasmid (GenePharma Inc. Shanghai, China) via EcoRI and BamHI restriction sites. The full length of human HMGN2 3′UTR and CDS regions were amplified by PCR from cDNA derived from human 293T cells. The PCR product was cloned into pmiReport luciferase reporter plasmid (Ribobio Inc. Guangzhou, China) via XhoI and NotI restriction sites. Assembly PCR was performed to mutate the 8 nucleotides of miR-23a seed region as indicated in Fig. 2A.

### Cell transfection

Double-strand miRNA mimic oligoribonucleotides for miR-155, miR-23a and their negative controls, single-strand miRNA inhibitor oligoribonucleotides for miR-155 and its negative control were synthesized in Ribobio Inc. (Guangzhou, China). siRNA for HMGN2 and its negative control were preserved in our laboratory^41^. The oligonucleotides or plasmids were transfected into A549 cells using Lipofectamine 2000 reagents per manufacturer’s instructions (Invitrogen, UnitedStates). The transfected cells werecultured for an additional 24 hours before they were harvested foranalysis.

### Bacterial adhesion assay

A549 cells and HBE16 cells (1 × 10^5 ^cells/well) were seeded into a 24-well plate and allowed to adhere overnight. Cells were infected by *K*. *pneumonia* at MOI = 100, Non-adherent bacteria were removed by washing with PBS for three times. 200 μl of 0.25% Triton X-100 was added to each well to lyse the cells for 15 min at 37 °C. Then cells were scraped, diluted, and plated onto LB agar plates. Colonies were counted to quantify the number of adherent bacteria.

### Luciferase Reporter Assay

For experimental validation of the HMGN2 3′ UTR as a target of miR-155 or miR-23a, co-transfections of reporter constructs and miR-155 (or miR-23a) mimic were carried out in A549 cell. After 24 hours of transfection, cells were lysed and luciferase activity was measured on 96-well black plates in a Microplate reader (Thermo, USA). Luciferase activities were measured by the relative activity of Renilla/firefly luciferase unit (RLU) using a Dual-Luciferase Reporter Assay (Beyotime Institute of Biotechnology, Haimen, China).

### Real-time quantitative polymerase chain reaction (RT-qPCR)

MiR-155, pri-miR-155, miR-23a, mRNA of HMGN2, integrin α5, integrin β1, NFIA and NFIB were investigated using RT-qPCR. Total RNAs were extracted using Total RNA Kit (OMEGA, USA). cDNA synthesis was achieved using the RevertAid First Strand cDNA Synthesis Kit (Thermo, USA). The sequence of the primer used for reverse transcription of mature miRNAsincluded a stem-loop structure. PCR products were detected with Thermo Scientific Maxima^®^ SYBR Green. The RT and PCR primers of miR-155, miR-23a and U6 (internal control of miRNA) were provided by Ribobio Inc. (Guangzhou, China). The primers were used as follows: pri-miR-155 (forward: 5′-GAC ACA AGG CCT GTT ACT AGC AC-3′, reverse: 5′-GTC TGA CAT CTA CGT TCA TCC AGC-3′); HMGN2 (forward: 5′-CCA TTG AAG AAGGGA GTT TGA-3′, reverse: 5′-ATC AGA GGC AGC ATT CCA AG-3′); integrin α5 (forward: 5′-TGC AGT GTG AGG CTG TGT ACA-3′, reverse: 5′-GTG GCC ACC TGA CGC TCT-3′); integrin β1 (forward: 5′-CTC AAG CCA GAG GAT ATT AC-3′, reverse: 5′-TCA TTG AGT AAG ACA GGT CC-3′); NFIA (forward: 5′-ACC CAG CAC ATC CTC TAC GA-3′, reverse: 5′-TGA CTG ACT GCC ACT TCC TG-3′); NFIB (forward: 5′-ACA AAG TCT GGC GTC TGG AT-3′, reverse: 5′-GGC TGG ACA CAA AGT GCT G-3′); GAPDH (forward: 5′-TGC ACC ACC AAC TGC TTA GC-3′, reverse: 5′-GGC ATG GAC TGT GGT CAT GAG-3′).

### Rac1-GTPase activity pull down assay

The activation of Rac1-GTPase was assessed using a pull-down assay kit (NewEast Bioscience). Briefly, A549 cells were lysed in ice-cold RIPA buffer with protease inhibitors. The samples were then incubated with the mouse monoclonal active-Rac1 antibody at 4 °C overnight. The next day, protein A/G agarose beads were added to incubate for 4 hours, then the beads were pelleted, and washed with RIPA buffer for three times, and then resuspended in 1 × SDS-PAGE protein loading buffer. The pull-down samples were heated to 95 °C for 5 min and resolved on 15% SDS-PAGE then detected by anti-Rac1 polyclonal antibody (1:1000).

### Western blotting (WB) assay

In Brief, Total protein was extracted using whole Cell Lysis Assay (Keygen Biotech Inc., Jiangsu, China). The protein concentration was carried out with a Thermo Scientific BCA protein assay kit (Rockford, USA). Cell lysates were heated to 95 °C for 5 min and then subjected to 15% SDS-polyacrylamide gel electrophoresis. Then the proteins were then blotted onto nitrocellulose membranes. Western blotting analyses were performed with the primary antibodies (mouse anti-F-actin, anti-Rac1, anti-GAPDH, anti-NFI and rabbit anti-HMGN2, anti-integrin α5, anti-integrin β1), followed by horseradish peroxidase-conjugated secondary antibody. Signals were detected by enhanced chemiluminescence reagent (Bio-Rad, USA).

### Fluorescence microscopy assay

Fluorescence microscopy was used to measure the membrane ruffles formed by polymerized F-actin and the expression of integrin α5 and β1. The A549 cells were seeded in the climbing pieces, after being transfected with miR-155 mimic or inhibitor, *K*. *pneumoniae* was added for 120 minutes at 37 °C. Subsequently, cells were washed twice with pre-cold PBS, then fixed in 4% PFA for 15 min and permeabilized with 0.1% Triton X-100 in PBS for 10 min at room temperature. Afterwards, For F-actin detection, cells were stained with 5 μg/ml rhodamine-phalloidin diluted in PBS for 60 min in the dark at 37 °C. For integrin α5 and β1, cells were incubated with antibodies of integrin α5 and β1 overnight, then stained with FITC-secondary antibody for 60 min in the dark at 37 °C. Cells were then washed, mounted, and visually examined with a Carl Zeiss Axio Scope A1 fluorescence microscope (Jena, Germany).

### Fibronectin (FN)-cell adhesion assay

FN-cell adhesion assay was assessed as previously described^35^. Briefly, 96-Well plates were coated with 100 μL (100 μg/mL) fibronetin or bovine serum albumin (BSA) overnight and 1% BSA was used to block nonspecific binding sites in the wells for 1 hour. Wells were washed with PBS. Treated cells were washed once and resuspended in serum-free RPMI 1640. Then 4 × 10^4^ cells per well were added to each plate. Cells were incubated for 1 hour at 37 °C and 5% CO_2_, washed with PBS twice, and were put back into 100 μL serum free media. Cell Counting Kit (Zoman, Beijing) was used to dye adhesion cells, plates were read at 540 nm on an automated microtiter plate reader (Thermo, USA). A blank well containing only media was also run as a control in all experiments.

### Chromosome Immunoprecipitation Assay (ChIp)

ChIp analyses were conducted on A549 cells according to manufacturer’s protocol (SimpleChIP^®^ Enzymatic Chromatin IP Kit (Magnetic Beads), Cell Signaling Technology Inc. Danvers, USA) with antibodies against NFI. The resultant DNA was analyzed by RT-qPCR using a pair of primers spanning the integrin α5 and β1 gene promoter (Fig. 5D). The primers were used as follows: integrin α5 gene promoter (forward: 5′-CTC AGA GTT CCA GGG ACC CA-3′, reverse: 5′-AAA CCT CCC AGA GGC GAA TG-3′); integrin β1 gene promoter (forward: 5′-CTT GCA GGA GAT TAG GGA CTG-3′, reverse: 5′-CTC ATT TCC TAG AGG TCT TCA GAT-3′).

### Statistical analysis

Data were expressed as mean values ± standard deviation (SD). All data analysis was tested by one-way analysis of variance for multiple comparisons with the LSD-test (homogeneity of variance) and Tamhane’s T2-test (heterogeneity of variance). p < 0.05 was considered to be had statistical significance.

## Additional Information

**How to cite this article**: Teng, Y. *et al*. The modulation of MiR-155 and MiR-23a manipulates *Klebsiella pneumoniae* Adhesion on Human pulmonary Epithelial cells via Integrin α5β1 Signaling. *Sci. Rep.* **6**, 31918; doi: 10.1038/srep31918 (2016).

## Supplementary Material

Supplementary Information
