# Supplementary material for: The modulation of MiR-155 and MiR-23a manipulates Klebsiella pneumoniae Adhesion on Human pulmonary Epithelial cells via Integrin α5β1 Signaling
Source: Sci Rep. 2016 Aug 18;6:31918. doi: 10.1038/srep31918 (PMC4989230; doi:10.1038/srep31918)

The modulation of MiR-155 and MiR-23a manipulates *Klebsiellapneumoniae* Adhesion on Human pulmonary Epithelial cells via Integrin α5β1 Signaling

Yan Teng[1a], Junming Miao[1a], Xiaofei Shen[1], Xiaolong Yang[1], Xinyuan Wang[1], Laibing Ren[1], Xiaoying Wang[1], Junli Chen[1], Jingyu Li[1], Shanze Chen[1], Yi Wang[1]*, Ning Huang[1]*

1 Research Unit of Infection and Immunity, Department of Pathophysiology, West China College of Basic and Forensic Medicine, Sichuan University, Chengdu 610041, China

* These authers are both Corresponding authors.

[a] These authors contributed equally to this work.

Tel: +86 02885501243;

E-mail:huangpanxiao@sina.com (Ning Huang).wangyi@stu.scu.edu.cn (Yi Wang)

FigS1. Relative expression of miR-155 and pri-miR-155 under *K. pneumoniae*or LPS stimulation.

A549 and HBE16 cells were exposed to increasing doses of *K. pneumoniae (*MOI=0, 50, 100) for 2 hours, or fixed amount of bacteria (MOI=100) at indicated time points (0hr, 2hr, 4hr and 6hr). The expression levels of miR-155 (A)and pri-miR-155 (B and C) were examined by RT-qPCR. RAW264.7 and A549 cells were stimulated by LPS (10 ng/mL) or exposed to fixed amount of bacteria (MOI=100) at indicated time points (0hr, 2hr and 6hr), the expression level of miR-155 (D and E) was examined by RT-qPCR. The Relative expression was normalized to U6 and then converted to the fold change over uninfected or unstimulated. (Data are the mean ± SD and represent three individual experiments. *p<0.05, **p<0.01 compared with *K. pneumoniae*or LPS unstimulated).

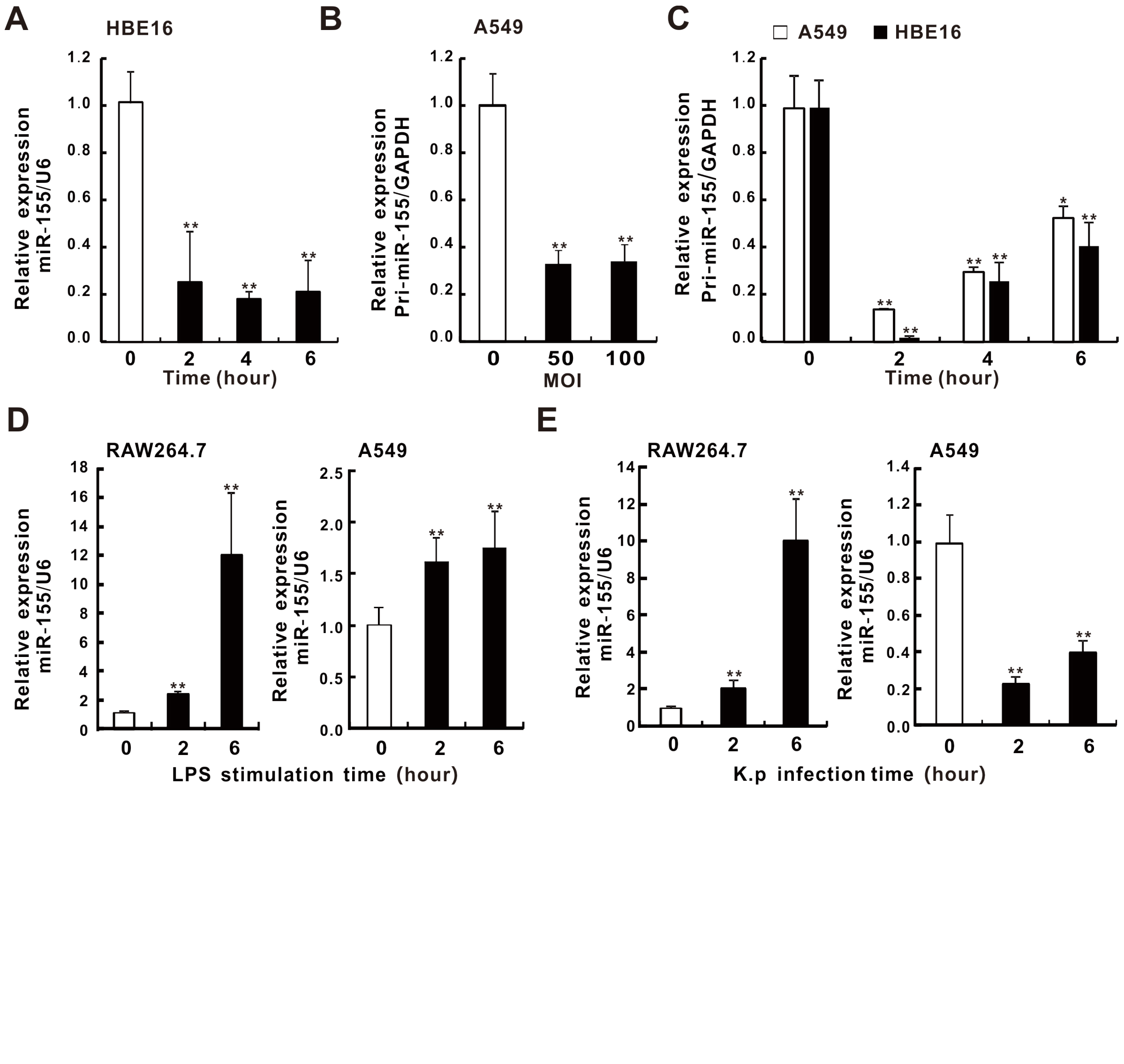

FigS2. MiR-155 regulated the adhesion of *K. pneumoniae*.

HBE16 cells were transfected with miR-155 mimic (A), inhibitor (B) and according negative control (miR-NC or NC) 24 hours prior to 100 MOI of *K. pneumoniae*exposure. The relative *K. pneumoniae* adhesion at indicated time points was determined by colony counts and represented after normalizing to 1 hour bacterial adhesion of miR-NC or NC. Relative expression of indicated miRNA after transfecting miR-155 mimic (C), inhibitor (D) or miR-23a mimic (E) in A549 or HBE16 cells (Folds over miR-NC or NC). (Data are the mean ± SD and represent three individual experiments. *p<0.05, **p<0.01).

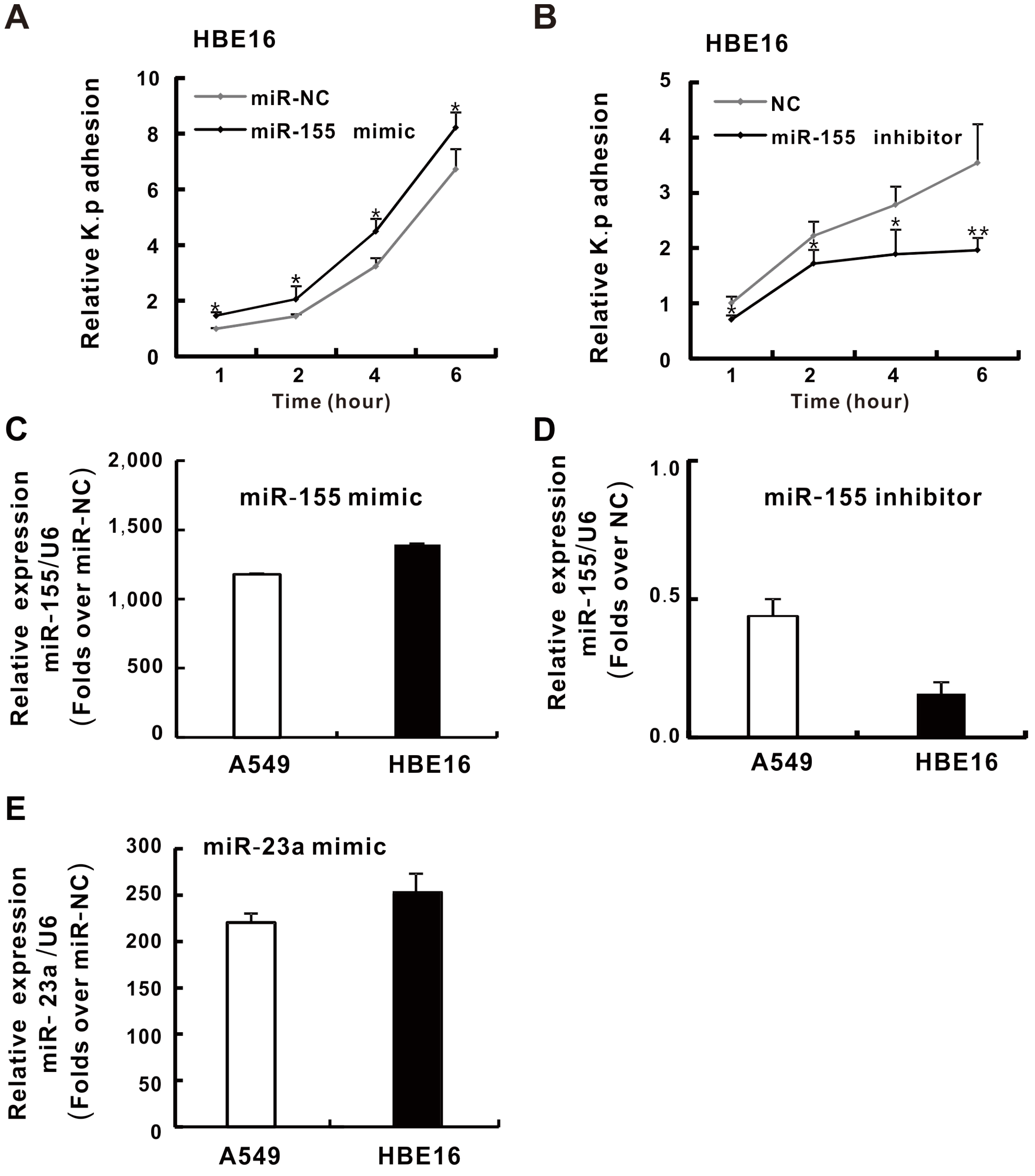

FigS3. HMGN2 was potential target of miR-155 and miR-23a.

(A) RT-qPCR analysis showing the change of HMGN2 mRNA level in *K. pneumoniae* infected A549 or HBE16 cells at different infection time points (MOI=100). (B) RT-qPCR analysis showing the effect of miR-155 mimic or inhibitor on the mRNA levels of HMGN2 in *K. pneumoniae* infected A549 and/or HBE16 cells (MOI=100 for 2 hours, same as B, C, D, E, and F). (C)A549 cells were co-transfected with miR-155 mimic or inhibitor and HMGN2 3' UTR wild-type (WT) luciferase reporter construct. Luciferase activity was measured 24hr after transfection. (D) A549 cells transfected with miR-155 mimic were treated with proteasome inhibitor (MG132, 20 μM, 3 hours) the relative HMGN2 protein expression were assessed by Western blot. (E) HMGN2 protein levels of A549 cells transfected with pEx-HMGN2 or si-HMGN2 (Data are the mean ± SD and represent three individual experiments. **p<0.01).

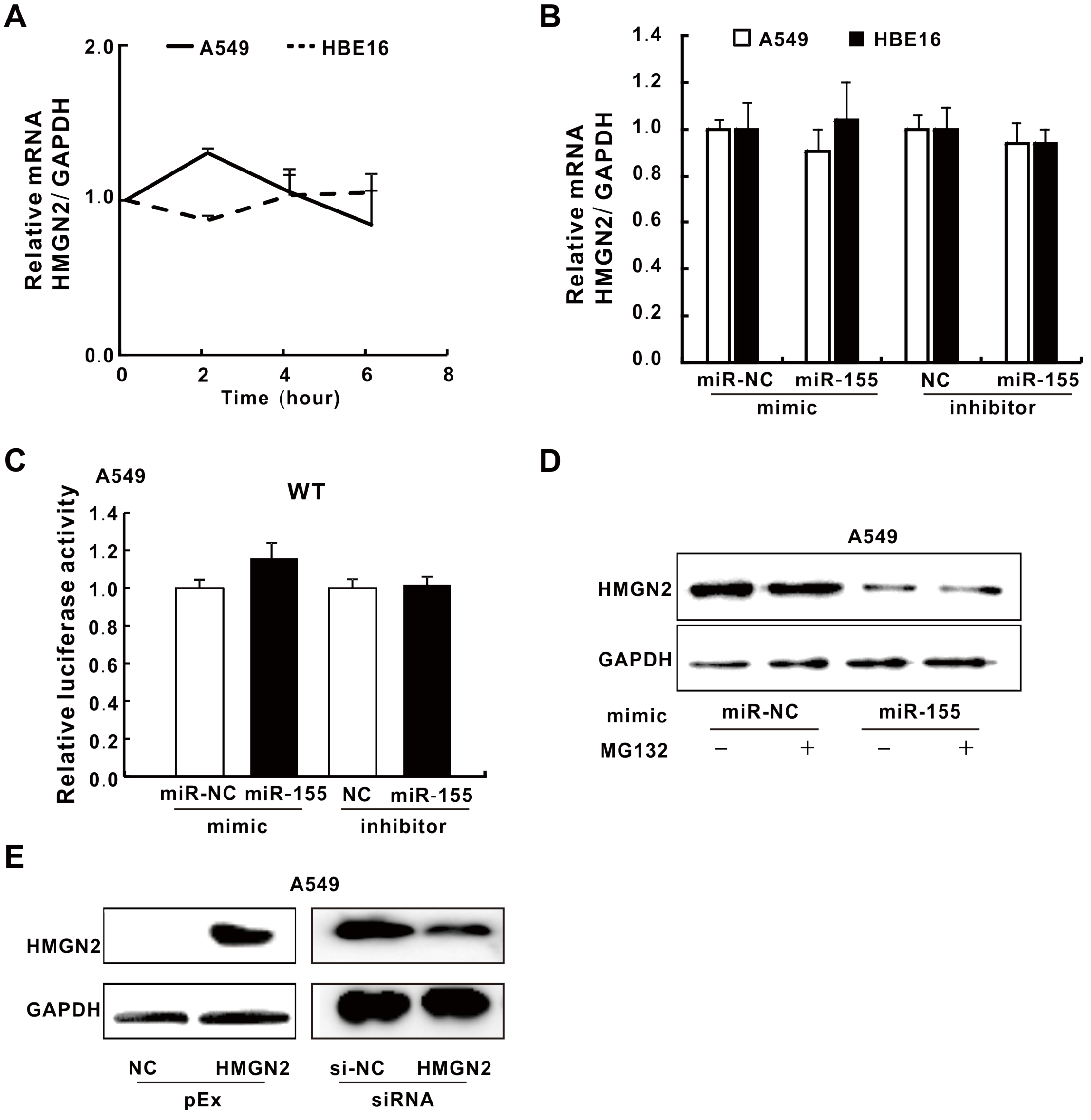

FigS4. MiR-155 promoted integrin-Rac1 pathway in *K. pneumoniae*-infected HBE16 cells.

(A) RT-qPCR analysis showingthe integrin α5 and β1 mRNA expression of HBE16 cells transfected with miR-155 mimic or miR-NC prior to *K. pneumoniae* infection (MOI=100 for 2 hours, same as B, D and E). Western blot analysis showing the levels of integrin α5, integrin β1(B), Rac1-GTP, total Rac1 (D) and F-actin (E) in HBE16 cells treated as (A).(C) FN–cell Adhesion assay was performed to evaluate the effect of miR-155 mimic on HBE16 cells to associate with fibronectin coated plates. (Data are the mean ± SD and represent three individual experiments. *p<0.05, **p<0.01 compared with miR-NC).

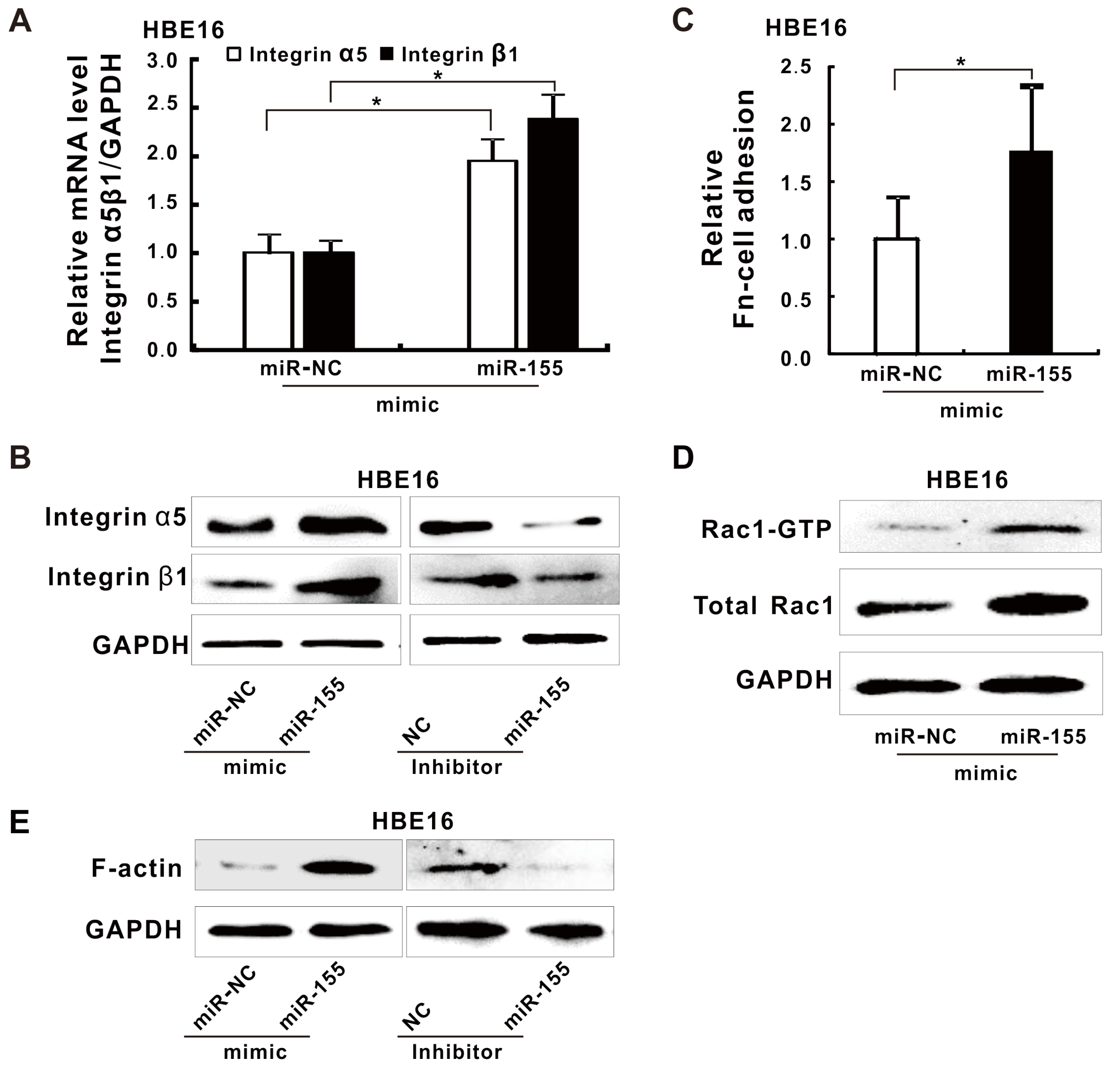

Supplement: Supplementary Information [file srep31918-s1.pdf]
